# Scientific Items

**Published:** 1886-01-20

**Authors:** 


### SCIENTIFIC ITEMS.

**The** (Popular Science News) recent explosion at Hell Gate was not only interesting as being one of the greatest submarine blasts ever attempted, but also presented some new scientific features. An explosive named rackrock was used to some extent in place of dynamite. This explosive consists of two ingredients—chlorate of potash and di-nitro benzole—neither of which is explosive alone, but which can be mixed together in such quantities as may be desired for immediate use, forming a compound but little less powerful than nitro-glycerine. In firing the mine, only a small proportion of the cartridges were exploded directly by the electric current; the concussion resulting from these proving sufficient to explode the remaining ones. A multiplicity of wires and detonators was thus avoided. It was intended to take advantage of the explosion to measure the rate at which the vibrations were transmitted through the earth. Numerous observers were stationed at points within a radius of a hundred miles, with seismographs and chronographs, ready to record the exact time of any unusual tremor of the earth. Unfortunately, the blast was delayed a short time; and most of the observers, considering that it had already taken place, had removed their instruments. Professor Young, at Princeton, fifty miles distant, obtained an approximate observation, which showed that the shock traversed the distance in about ninety seconds, or at the rate of nearly two thousand miles an hour. These observations have an important bearing on the subject of earthquake shocks, and the delay in firing the blast was very unfortunate.

**M Pasteur** (Popular Science News) announces that he has discovered a method of inoculation against hydrophobia which not only secures against the liability to contract the disease from bites by rabid animals, but also prevents the development of the disease in persons already bitten. He is said to have already operated on several persons who have been bitten by mad dogs, with great success; though how the "success" of the operation can be determined until considerable time has elapsed, is by no means clear. The accounts that have reached this country concerning the process are rather vague; but it appears to consist in inoculating the patients with portions of the spinal marrow of rabbits which in their turn have been subjected to the influence of hydrophobic virus of varying degrees of intensity. This matter is claimed to produce a mild attack of rabies in the individual, protecting him from the effects of the more powerful poison of rabid animals in the same manner that the mild virus of cow-pox protects the system against its dreaded relative, the small-pox.

**Inoculation in Yellow Fever.**—(Popular Science News) M. Bouley recently read a note on "Prophylactic Inoculation against Yellow Fever as practiced in Rio de Janeiro" at the Academy of Sciences, Paris. This experiment, first introduced by Dr.

Domingos Freire, has since been carried out on a large scale under the control of the government. Since the month of March, 1883, as many as eleven hundred and nine persons, of all ages, nationalities, and conditions of life, have been subjected to subcutaneous injections with the attenuated virus cultivated for the purpose. In some cases the injections were administered in houses where the scourge had a few hours before proved fatal to some of the inmates, yet no misadventure of any kind has followed; and this preventive measure seems, so far, to have been attended by the best results.

**Resisting Electric Shocks.**—(Popular Science News) Dr. A. L. Hummel recounts some curious cases of recovery from shocks, which ought, according to all ideas upon the subject, to have proved fatal. One is that of an employe of the Brush Company, who, while still grasping the wire in his left hand, cut it with a pair of nippers held in the right. The current was at once established through his body; and he was held to the wire at the top of a thirty-foot pole for three minutes at least, by which time a ladder had been procured, and he was released by a fellow-workman. During this time a current of three thousand volts, or sufficient to run fifty arc-lamps of two-thousand-candle power each, had apparently passed through his body; yet he was able to reach the pavement with slight assistance, suffered little or no constitutional disturbance, save a slight rise in temperature, and was not confined to his bed. His punishment was limited to the charring of three fingers on the left hand, and a small but deeply burned hole in the palm of the right hand. The only treatment was that applied for simple burns. Another case was afforded by one of the workmen of the Bell Telephone Company, who, having climbed a pole belonging to the Brush Company in order to carry a wire over it, grasped the tie-wire of the positive line with one hand, and that of the negative line with the other. Here he was held until an assistant ran five squares to the electric-light station, when the circuit was broken, and he was released. A rope had been attached to him, and passed over the arm of the pole. This was held by another man, and he was thus prevented from falling. Though unconscious for a while, he soon rallied; but his pulse never exceeded 86, nor his temperature 101°. The thumb of the left hand was burned nearly off, as were also the index and middle fingers of the right hand. These were all amputated.

**The "Big Woods" of Minnesota.**—These forests are rightly named; for they cover 5,000 square miles, or 3,200,000 acres of surface. These woods contain only hard-wood growths, including white and black oak, maple, hickory, basswood, elm, cottonwood, tamarack, and enough other varieties to make an aggregate of over thirty different kinds. The hard-wood tract extends in a belt across the middle of the State; and surround its north-eastern corner is an immense pine region covering 21,000 square miles, or 13,440,000 square acres.

